# OUTCOMES OF REHABILITATION FOLLOWING DEVICE-AIDED THERAPY START IN PARKINSON’S DISEASE: A RANDOMIZED CONTROLLED TRIAL

**DOI:** 10.2340/jrm.v58.46068

**Published:** 2026-08-03

**Authors:** Ann BJÖRKDAHL, Annika KHALIF, Marie GUSTAFSSON, Ann-Charlotte LINDSTRÖM, Filip BERGQUIST

**Affiliations:** 1 Department of Clinical Neuroscience, University of Gothenburg, Gothenburg; 2 Rehabilitation Medicine, Sahlgrenska University Hospital, Gothenburg; 3 Department for Occupational Therapy and Physiotherapy, Sahlgrenska University Hospital, Gothenburg; 4 Primary Care Rehabilitation, Västra Götaland; 5 Neurology, Sahlgrenska University Hospital, Gothenburg Sweden

**Keywords:** Parkinson’s disease, rehabilitation, device-aided therapy, deep brain stimulation, levodopa intestinal infusion, apomorphine subcutaneous infusion

## Abstract

**Objective:**

To determine whether adding tailored primary care rehabilitation to Device-Aided Therapy in Parkinson’s disease improves outcomes more than Device-Aided Therapy alone.

**Design:**

Open randomized controlled intervention trial.

**Subjects/Patients:**

Parkinson’s patients initiating Device-Aided Therapy.

**Methods:**

Participants were randomized to receive standard care either with or without 3 months of tailored primary care rehabilitation. The primary outcome was Assessment of Motor and Process Skills, while secondary outcomes included cognitive measures and overall treatment efficacy.

**Results:**

The study was completed by 45 of 54 included patients. The primary outcome variable indicated that rehabilitation resulted in better maintenance of patient motor skills at 6 and 12 months. However, among several secondary measures, only perceived health improved more in the intervention group. Motor fluctuations improved in both groups and several other disease-specific scales demonstrated non-significant improvements. The rehabilitation group showed better cognitive performance at 12 months.

**Conclusion:**

Motor skills and perceived health were improved by a 3-month rehabilitation period after the start of Device-Aided Therapy for advanced Parkinson’s disease. An observed positive effect of rehabilitation on cognition in the first year after the therapy requires further confirmation.

## LAY ABSTRACT

This study evaluated whether adding a 3-month personalized rehabilitation programme helps people with Parkinson’s disease starting advanced Device-Aided Therapy. Fifty-four patients were randomized to receive standard care either with or without 3 months of tailored primary care rehabilitation. Patients receiving rehabilitation maintained movement abilities better at 6 and 12 months compared with the standard treatment group. They also reported better perceived health over the first 6 months and demonstrated better cognitive function at 1 year. Starting Device-Aided Therapy was associated with more stable motor symptom relief and positive clinical trends in both groups. Because participants knew their group assignment, placebo effects may have influenced the results. In conclusion, this study shows that while Device-Aided Therapy is associated with improvements on its own, adding personalized rehabilitation may help Parkinson’s patients conserve motor skills and experience better health.

With time, approximately 10–30% of patients with Parkinson’s disease (PD) develop medication-related symptom fluctuations that are not sufficiently controlled with conventional treatments (1, 2), but can be improved with continuous, device-aided treatments (DATs), specifically deep brain stimulation (DBS) or dopaminergic infusion therapies including continuous subcutaneous apomorphine infusion (SCAI) and intestinal or subcutaneous infusion with levodopa or foslevodopa (3, 4). The timing of these DATs, in particular DBS, has been contested (5), and earlier introduction has been proposed (6, 7). Currently it is, however, common that DAT is introduced only after some impairment of daily activities and health-related quality of life (HRQoL) has developed. Most patients have therefore ceased some activities because the fluctuations make it difficult to maintain them. Once a change in behaviour is established it is not self-evident that improving fluctuations will be enough to regain activities. One example is the degree of workforce participation, which does not seem to improve even after early DBS introduction despite improvement in many symptoms and in HRQoL (8). Similarly, an observational study showed that workforce participation may be maintained, but rarely increased, after DAT introduction (9). Research has shown that when people lose the ability to perform tasks that are important to them, their perception of themselves as capable and competent disappears, leading to a sense of being worthless and meaningless (10). Reducing symptom fluctuations may, although beneficial overall, not be enough to regain capabilities when habits and self-perception have changed. Appropriate interventions may be needed to help the individual resume meaningful activities that can contribute to better health and well-being. Although all DATs have demonstrated clinically meaningful effects, health economic benefit is best established for deep brain stimulation (11). DATs are costly, but infusion therapies incur higher costs over time, amounting to around €80 per day. It is therefore important to get the best possible efficacy from treatment. Early rehabilitation is one such strategy, but the evidence for this is limited to open uncontrolled studies and case reports after DBS (12). Investigating the effect of early rehabilitation after DAT introduction is therefore an unmet need and the result of such studies could guide clinical practice to optimize the effectiveness of DAT in PD patients with uncontrolled symptom fluctuations.

We hypothesized that active rehabilitation may improve the effect of symptom stabilization after DAT, and that this would increase activity, resulting in better ADL performance as well as autonomy and confidence. To investigate the possibility that dedicated rehabilitation improves the outcome of DAT in PD patients with symptom fluctuations, we randomized patients who were about to start treatment with DBS of the subthalamic nuclei (STN-DBS), SCAI, or intestinal continuous levodopa infusion with or without entacapone (ICLI), to either standard care plus a 3-month individualized rehabilitation programme, or standard care. The primary outcome was a standard assessment of motor skills, and secondary outcomes included standard assessment of process skills, patient-reported health-related quality of life, measures of participation and autonomy, fatigue and confidence in balance, as well as disease-specific symptom rating scales and cognition.

## Methods

### Design and sample

**Fig. 1:**
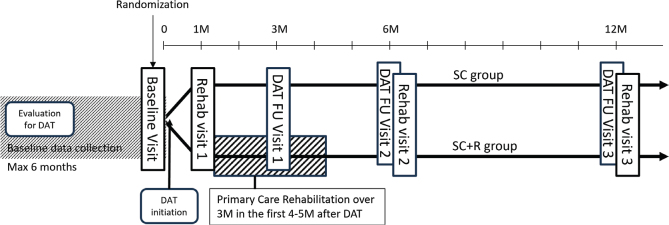
Outline of study design. DAT: Device-Aided Therapy; FU: follow-up; SC: standard care; SC+R: standard care plus rehabilitation.

This single-centre randomized controlled study enrolled PD patients initiating DAT for motor fluctuations between 2016 and 2021. DAT comprised subthalamic nucleus DBS, continuous intestinal levodopa/carbidopa or levodopa/entacapone/carbidopa infusion (ICLI), or subcutaneous apomorphine infusion (SCAI). Patients were randomized to standard care alone (SC) or to standard care plus a 3-month personalized rehabilitation programme (SC+R), with rehabilitation commencing 1 month post-DAT. DAT initiation was defined as first DBS activation, PEG placement for ICLI, or SCAI start. Outcomes were assessed at baseline (pre-DAT) and at 1, 3, 6, and 12 months post DAT initiation (Fig. 1). The study was conducted in collaboration between the Neurology clinic and the Rehabilitation Medicine clinic at Sahlgrenska University Hospital and primary care rehabilitation units in Västra Götaland/Halland, Sweden.

The inclusion criteria were PD diagnosis as per UK PD Society Brain Bank Clinical Diagnostic Criteria (13), residency in Västra Götaland or Halland, capacity for active rehabilitation, and willingness to comply with the protocol including keeping an activity diary. Patients were excluded if they had pre-existing conditions substantially limiting rehabilitation, were undergoing active primary care rehabilitation, or could not follow instructions. At the time of planning the study, there was limited information on Assessment of Motor and Process Skills (AMPS) performance in PD patients but, based on a clinically important effect of 0.5 and reported Process skill scores in a PD population (14), a sample size of 30 was estimated for that measure (type 1 error 5%, power 0.8, mean (SD) 0,9 (0.55). For AMPS Motor skill we assumed the mean would be close to the threshold of independence in ADL (1.5), and that SD would be proportional to the SD for Process skills. This resulted in a calculated sample size of 82.

### Randomization

Randomization was performed at the baseline neurology visit using the adaptive Qminim online minimization tool (15), balancing groups on: sex, age (20–49/50–69/ ≥ 70), DAT modality, disease duration (3–9/10–14/ ≥ 15 years), and MoCA score (0–18/19–25/26–30). The first participant was randomized simply; subsequent participants were assigned using the Pocock–Simon marginal balance algorithm (probability 0.7 for the minimizing arm).

### Follow-up and outcome assessments

Medical follow-up occurred at 3, 6, and 12 months as per standard local practice. Rehabilitation outcome assessments began 1 month after DAT initiation and were repeated at 6 and 12 months; assessment procedures were identical for both groups.

### Intervention

SC+R began with a 2-day multidisciplinary evaluation at the Sahlgrenska Rehabilitation Clinic to develop an individualized rehabilitation plan, centred on the patient’s own goals. The plan was communicated to the patient’s local primary care rehabilitation team, which typically comprised occupational therapists, physiotherapists, and speech therapists from a specialized neuro care unit; standard primary care resources were used where specialist neuro teams were unavailable. Patients were to receive a minimum of 4 visits each to a physiotherapist and occupational therapist (and speech therapist where indicated) over 3 months. The overarching aim was to maximize functional benefit from DAT, optimize quality of life, and preventively address long-term disease management.

### Standard care

Standard care involved regular post-DAT follow-up with treatment adjustments as clinically indicated. Rehabilitation was not prohibited and could be provided if deemed necessary. Upon completing 12-month follow-up, control participants were offered the 3-month rehabilitation programme.

### Primary outcome

*Assessment of Motor and Process Skills (AMPS).* The AMPS (16, 17) is an observational assessment of 16 motor and 20 process skills during instrumental ADL activities. Performance on 2 standardized tasks is rated on a 4-point Likert scale; many-faceted Rasch analysis converts scores to logit measures adjusted for task challenge, item difficulty, and rater severity. Thresholds for independent ADL are 1.5 logits (motor) and 1.0 logits (process); a 0.5-logit change is clinically meaningful. Unlike commonly used ADL instruments such as the Barthel Index and Functional Independence Measure, AMPS assesses actual task performance rather than degree of independence, providing a more sensitive and ecologically valid measure (16). As both DAT and most rehabilitation of PD patients focus on motor symptoms, AMPS motor skill was selected as the primary outcome of the study, with the null hypothesis that there would be no difference between the groups regarding AMPS motor skill after the rehabilitation period. To account for possible baseline differences, the baseline AMPS motor skill assessment was used as a covariate.

### Secondary and exploratory outcomes

*AMPS.* AMPS process skill was considered a secondary outcome. Furthermore, the proportion of patients performing worse than the threshold for independent ADL (motor and process skill) was an exploratory outcome.

*Falls Efficacy Scale (FES).* 13-item questionnaire (0–10 per item) assessing balance confidence; higher scores indicate greater confidence (18).

*Mental Fatigue Scale (MFS).* 14 items (0–3 scale) evaluating mental fatigue; scores > 10 indicate significant brain fatigue (19).

*Impact of Participation and Autonomy (IPA).* Assesses perceived autonomy and participation across 5 domains (autonomy indoors, family role, autonomy outdoors, social relations, work/education; 31 items in total, scored 0–4); lower scores indicate better participation (20, 21).

*PDQ-8.* Eight-item version of the Parkinson’s Disease Questionnaire (22). Items rated 0–4; scores converted to a summary index (PDQ-8-SI, 0–100) reflecting percentage of maximum problems.

*EQ-5D-5L.* Five-dimension health measure (mobility, self-care, usual activities, pain/discomfort, anxiety/depression) plus a visual analogue scale (EQ-VAS) (23). Summary index calculated using the 2022 Swedish value set.

*MDS-UPDRS.* Clinician-administered and patient-reported scale across 4 parts (non-motor ADL, motor ADL, motor examination, motor complications); total score 0–260, higher scores indicate greater disease burden (24).

*MADRS-S.* Nine-item self-report depression measure (0–6 per item; total 0–54); higher scores indicate greater severity (25).

*Parkinson Kinetigraph (PKG).* Wrist-worn actigraphy device recording data continuously for 6–10 days (26), generating scores for bradykinesia, dyskinesia, tremor, fluctuations, and sleep. Baseline PKG was obtained within 6 months prior to DAT; follow-up measurements were aligned with neurology clinic visits.

*Non-Motor Symptom Questionnaire (NMSQUEST).* 30-item Yes/No screening tool for non-motor symptoms across multiple domains (27); total score (0–30) reflects the number of symptoms present.

*Montreal Cognitive Assessment (MoCA).* 30-point clinician-administered cognitive screening tool; scores ≥ 26 are considered normal (28). Assessed pre-DAT or at baseline and repeated at month 12.

*Activity log.* Both groups recorded daily activity (work, household, physical training, other physical activity, rest) in the 2 months following the first rehabilitation assessment visit. Data were collected daily for the first 2 weeks, then over 4-day intervals (Thursday–Sunday) in week seven and twelve.

### Safety

Reporting of rehabilitation-related adverse events were encouraged to take place at all follow-up visits.

### Data analyses

Descriptive analysis of demographic and clinical characteristics was performed for all randomized and all completing patients. All available data from patients with at least 1 post-intervention assessment were analysed. No permutations of missing data were made. Baseline characteristics are presented as mean±SD; between-group differences in continuous variables were assessed using independent *t*-tests with bootstrapped 95% CIs and *p*-values (1,000 samples) to account for small sample size and potential non-normality. Categorical variables were compared using the Hauck–Anderson test or the Fisher–Freeman–Halton exact test. Effect sizes are reported as Cohen’s *d* (small: 0.2, medium: 0.5, large: 0.8). Intervention content was described in terms of visit frequency and rehabilitation activities. Outcome data are presented as mean±SD (parametric), median and range (ordinal), or percentages.

Longitudinal intervention effects were analysed using a repeated-measures linear mixed model restricted to the post-randomization time points, with timepoint, randomization group, and their interaction as fixed effects. The baseline assessment was used as a covariate together with the variable that differed between groups at randomization (living with or without a partner) and the minimizing factors. Before analysis, continuous baseline covariates were standardized (z-scores), and larger scale outcome variables were scaled down by a factor of 10 or 100 as appropriate. An unstructured covariance matrix accommodated heterogeneous variances and within-person correlations across timepoints. Models were estimated by restricted maximum likelihood; degrees of freedom were computed via Satterthwaite’s approximation. An exploratory generalized estimating equation analysis was used to examine the proportion of patients performing below AMPS autonomy thresholds over time. Significant main effects and interactions were followed by Bonferroni-corrected *post-hoc* comparisons. For outcomes that had a pre-DAT baseline (MDS-UPDRS, PDQ8-SI, NMSQ, MADRS-S, and EQ5D5L), the intercepts of the linear mixed model were compared with the baseline means pooled from both groups using 95% confidence intervals. The significance threshold was α = 0.05.

## Results

**Fig. 2:**
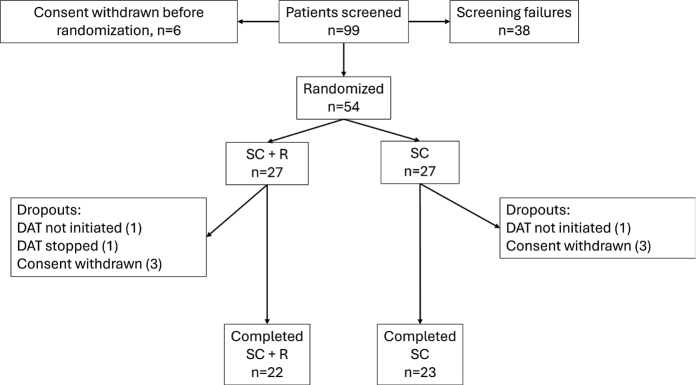
Participant flow through the phases of the randomized controlled trial. Of 99 patients screened, 54 met the eligibility criteria and were randomized 1:1 into the Standard Care plus Rehabilitation (SC + R, *n* = 27) and Standard Care (SC, *n* = 27) groups. Reasons for attrition, including screening failures and dropouts during the study period, are specified at each stage. DAT: Device-Aided Therapy.

Over 5.5 years (2016–2021), 99 patients were screened for eligibility among those offered DAT at Sahlgrenska University Hospital. Of these, 38 were ineligible and 7 withdrew consent before randomization, yielding 54 randomized participants. The clinical and demographic characteristics did not differ between the randomization groups (Table I). Forty-five patients (83%) completed the study; the main reasons for dropout (5 from SC+R and 4 from SC) were withdrawal of consent and DAT not being initiated or being discontinued (Fig. 2). The study was terminated before inclusion of the planned 90 patients due to slow recruitment rate. No rehabilitation-related adverse events were reported.

**Table I:** Clinical and demographic characteristics of randomized patients

Item	Standard care (*n* = 27)	Rehab intervention (*n* = 27)	*p*-value^a^
Age at DAT start, mean ± SD, *n*	64 ± 9, 27	66 ± 9, 27	0.408
Disease duration, mean ± SD, *n*	14 ± 6, 27	13 ± 5, 27	0.570
Hoehn &Yahr stage, mean ± SD, *n*	2.2 ± 0.7, 25	2.2 ± 0.6, 26	0.870
MoCA, mean ± SD, *n*	25.3 ± 3.0, 27	24.5 ± 4.3, 27	0.419
Sex F/M, *n* (%F)	15/12 (55)	12/15 (44)	0.414
Planned DAT: DBS/ICLI/SCAI, *n* (%)	11/14/2 (41/52/7)	9/14/4 (33/52/15)	0.584^b^

a For continuous data: unpaired *t*-test, equal variances not assumed, for categorical data: Hauck Anderson independent sample proportion test.

b Fisher–Freeman–Halton exact test.

DAT: Device-Aided Therapy; MoCA: Montreal Cognitive Assessment; F: female; M: male; DBS: deep brain stimulation; ICLI: intestinal continuous levodopa infusion; SCAI: subcutaneous continuous apomorphine infusion.

The patients who completed the study in the 2 groups were well matched across continuous demographic and clinical variables (Table II; all independent *t*-tests *p* > 0.05, confirmed by bootstrapped 95% CIs and *p*-values). The proportion living with a partner was higher in SC+R than SC (bootstrapped *p* = 0.035). The distribution of implemented DAT modality did not differ between groups (Fisher–Freeman–Halton exact test, *p* = 0.372).

**Table II:** Clinical characteristics of patients completing the study

Item	Standard care (*n* = 23)	Rehab intervention (*n* = 22)	*p*-value^a^
Age at DAT start, mean ± SD, *n*	64 ± 9, 23	65 ± 9, 22	0.590
Disease duration, mean ± SD, *n*	15 ± 6, 23	14 ± 4, 22	0.591
Hoehn &Yahr stage, mean ± SD, *n*	2.2 ± 0.5, 22	2.2 ± 0.6, 22	0.847
MoCA, mean ± SD, *n*	25.2 ± 3.1, 23	25.0 ± 4.3, 22	0.848
MDS-UPDRS I, mean ± SD, *n*	12.0 ± 6.5, 22	12.9 ± 6.7, 22	0.664
MDS-UPDRS II, mean ± SD, *n*	19.4 ± 6.1, 22	18.9 ± 6.0, 22	0.785
MDS-UPDRS III, mean ± SD, *n*	34.5 ± 11.1, 22	29.8 ± 12.2, 22	0.186
MDS-UPDRS IV, mean ± SD, *n*	10.9 ± 3.0, 22	10.5 ± 2.8, 22	0.677
Sum MDS-UPDRS, mean ± SD, *n*	76.4 ± 18.8, 22	71.3 ± 19.4, 22	0.381
Bodyweight (kg), mean ± SD, *n*	66.9 ± 17.0, 20	75.5 ± 16.0, 17	0.118
PKG FDS, mean ± SD, *n*	12.1 ± 5.1, 23	11.8 ± 3.7, 22	0.834
PKG BK median, mean ± SD, *n*	21.6 ± 6.0, 23	23.4 ± 6.4, 22	0.332
PKG DK median, mean ± SD, *n*	9.2 ± 12.1, 23	5.1 ± 5.1, 22	0.150
PKG PTI%, mean ± SD, *n*	7.4 ± 7.7, 22	4.9 ± 4.0, 21	0.180
PKG PTT%, mean ± SD, *n*	5.4 ± 9.3, 22	5.0 ± 9.1, 21	0.908
PDQ8 SI, mean ± SD, *n*	44.0 ± 17.3, 22	42.1 ± 12.3, 20	0.682
MADRS-S, mean ± SD, *n*	10.6 ± 6.0, 20	12.3 ± 6.9, 16	0.435
EQ5D5L index, mean ± SD, *n*	0.588 ± 0.193, 22	0.585 ± 0.175, 19	0.961
EQ-VAS, mean ± SD, *n*	52.8 ± 16.5, 22	49.1 ± 20.5, 20	0.529
NMS Questionnaire, mean ± SD, *n*	12.4 ± 4.8, 20	12.7 ± 5.2, 20	0.852
Sex F/M, *n* (%F)	11/12 (48)	9/13 (41)	0.759
Living with partner, *n* (%)	13 (56)	19 (86)	**0.033**
≥ 12 years education, *n* (%)	19 (86)	19 (90)	0.862
> 50% work capacity, *n* (%)	3 (14)	2 (9)	0.779
Performed DAT: DBS/ICLI/SCAI, *n* (%)	10/12/1 (44/52/4)	8/10/4 (36/45/18)	0.372^b^

a For continuous data: unpaired *t*-test, equal variances not assumed, for categorical data: Hauck Anderson independent sample proportion test.

b Fisher–Freeman–Halton exact test.

DAT: Device-Aided Therapy; MoCA: Montreal Cognitive Assessment; MDS-UPDRS: Movement Disorder Society Unified Parkinson’s Disease Rating Scale; PKG: Parkinson kinetigraph; FDS: fluctuation dyskinesia score; BK: bradykinesia score; DK: dyskinesia score; PTI: percentage time with immobility; PTT: percentage time with tremor; PDQ8 SI: Parkinson Disease Questionnaire 8-question version Summary Index; MADRS-S: Montgomery–Åsberg Depression Rating Scale, self-administered; EQ5D5L: EuroQoL 5-dimension, 5-level scale; EQ-VAS: EuroQoLVisual Analogue Scale; NMS: non motor symptom; F: female; M: male; DBS: deep brain stimulation; ICLI: intestinal continuous levodopa infusion; SCAI: subcutaneous continuous apomorphine infusion.

Rehabilitation activities in SC+R are described in Table III and Fig. 6. Activity log data (Table IV) showed high inter-individual variability. Work activity was reported by only a few patients, who thereby contributed with many hours that did not primarily have a rehabilitating purpose. Work activity was therefore omitted from the analysis. No statistically significant between-group differences in total daily activity time or proportion with < 5 h of activity per week were found, but SC consistently showed a numerically considerably higher proportion (12.5% to 25%) below this threshold across all four recording occasions compared with SC+R (0% to 4.8%).

**Table III:** Rehabilitation activities of patients randomized to standard care plus rehabilitation

Profession	Median number of visits per patient (IQR)	Median duration of visits in minutes (IQR)	Typical activities
Physiotherapist	11 (7.8)	44 (9)	Advice and support, assessments, and training of motor skills and body function
Occupational Therapist	5 (2.5)	62 (22)	Advice and support, prescription of assistive devices and home adaptations, and assessment of activity performance
Speech Therapist	4.5 (8.0)	42 (13)	Assessment of voice and speech, advice, support and planning, and speech therapy

IQR: iinterquartile range.

**Table IV:** Diary-registered activity time

Activity	Group	Week 1	Week 2	Week 7	Week 12
h/day Median (QL, QU)	SC+R	2.2 (1.6, 3.5)	2.8 (2.2, 3.4)	2.7 (1.9, 4.4)	2.3 (1.4, 4.0)
	SC	1.9 (0.7, 3.4)	1.6 (0.9, 3.1)	2.3 (1.3, 4.0)	1.4 (0.6, 3.3)
< 5 h activity/week *n* (%)	SC+R	1 (4.8%)	1 (4.8%)	0 (0%)	1 (4.8%)
	SC	6 (25%)	4 (16.7%)	3 (12.5%)	6 (25%)

QL: lower quartile; QU: upper quartile; SC: standard care; SC+R: standard care plus rehabilitation.

### Primary outcome

**Fig. 3:**
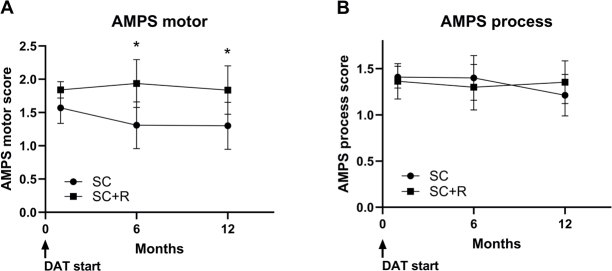
Longitudinal change in AMPS scores. Longitudinal change in estimated means of (A) AMPS motor skills score and (B) AMPS process skills score. Month 1 data are mean and months 6 and 12 estimated means. Error bars indicate 95% confidence intervals. Data were analysed with a linear mixed model with the month 1 baseline, minimizing factors and living condition as covariates. SC: standard care; SC+R: standard care plus rehabilitation.

The linear mixed model analysis revealed a significant main effect of rehabilitation group on AMPS motor skills scores (*F*[1, 43.2] = 6.01, *p* = 0.018, with an estimated mean for the SC group of 1.30, CI_95_ 0.97–1.64 and the estimated mean for SC+R was 1.89, CI_95_ 1.54–2.23), with no significant effect of time and no group × time interaction. *Post hoc* analysis indicated significant differences at both 6 and 12 months in favour of the rehabilitation intervention (Fig. 3A), with effect sizes of 0.75 at 6 months and 0.64 at 12 months (Cohen’s *d*).

### Secondary outcomes

No significant effects were found for AMPS process skills, FES, MFS, or any IPA domain (Fig. 3B; Table SI).

Cognitive function developed somewhat differently between groups over 12 months: MoCA scores increased by 1.4 (± 2.3) points for the SC+R group and decreased by 0.3 (± 3.1) points in the SC group (mean difference in change 1.8 points, 95% CI [0.07, 3.45]; independent *t*-test, *p* = 0.041; Cohen’s *d* = 0.65, 95% CI [0.03, 1.27]).

**Fig. 4:**
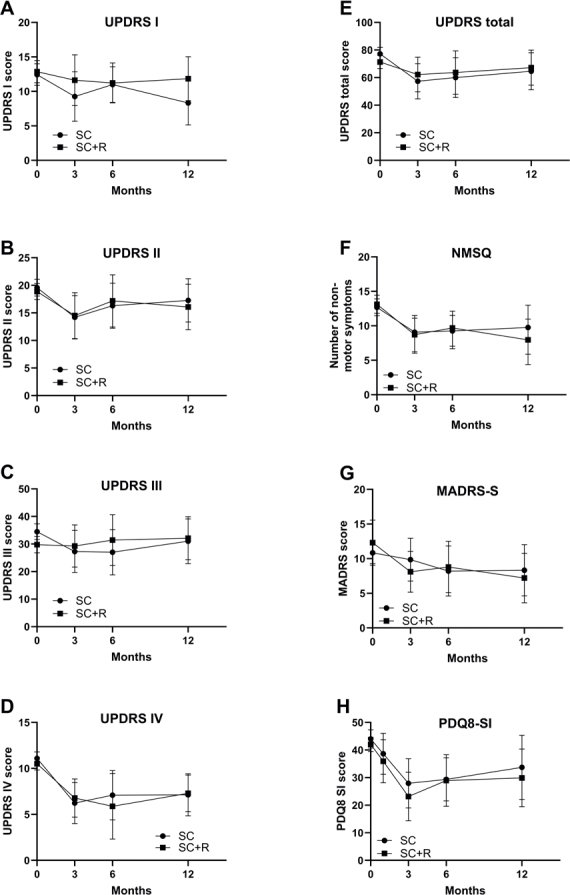
Longitudinal changes in disease-specific and depression ratings. (A–D) Longitudinal changes in estimated means (EM) of the Movement Disorder Society unified Parkinson’s disease rating subscales MDS-UPDRS I–IV and (E) the sum of MDS-UPDRS subscales. (F) EM of number of reported non-motor symptoms over time NMSQ and (G) EM of Montgomery–Åsberg Depression Rating Scale, self-assessment version, MADRS-S. (H) EM of Parkinson’s Disease Quality of life 8 question version summary index. Baseline values represented as mean and post baseline values as estimated means. Error bars indicate 95% confidence intervals. SC: standard care; SC+R: standard care plus rehabilitation.

**Fig. 5:**
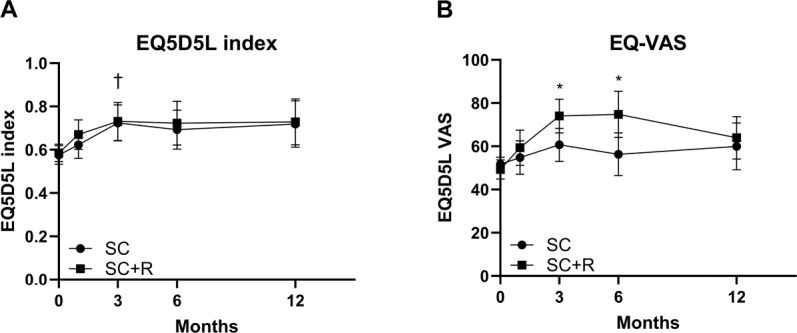
Longitudinal changes in EuroQoL quality of life measures**. (A)** Longitudinal changes in estimated means (EM) of the EuroQoL 5 domain, 5 level index (EQ5D5L index), and (B) EM of the visual analogue scale of perceived health (EQ-VAS). The dagger indicates a significant difference compared with the 1-month time point and the asterisks indicate significant differences between groups in *post-hoc* tests. SC: standard care, SC+R: standard care plus rehabilitation.

There was no time effect, randomization group effect, or interaction in the linear mixed models for the PD-specific scales MDS-UPDRS and NMSQ at time points after the start of DAT (Fig. 4). However, the model intercept values were lower than the pre-DAT baselines for NMSQ, MDS-UPDRS I, II, IV and the total MDS-UPDRS score. For MDS-UPDRS IV, the overall estimated mean (intercept) of post-DAT time points had a 95% confidence interval that did not overlap the confidence intervals for the pooled pre-DAT mean (7.3, 5.3–9.3, vs 10.8, 10.1–11.6, Fig. 4D). The disease-specific quality of life scale PDQ8-SI and the depressive symptoms scale MADRS-S both displayed a significant effect of time over the post-DAT initiation period, together with lower model intercepts than baseline means, but with overlapping confidence intervals (MADRS-S baseline: 11.5, 9.4–13.6, model intercept 7.2, 3.3–11.1; PDQ8-SI baseline: 43, 39–48, model intercept: 30, 19–40, Fig 4G, H). EQ-5D-5L index increased with time post DAT (*F*[3, 18.4] = 3.89, *p* = 0.026) with an increase from the estimated mean (CI_95_) 0.646 (0.600–0.693) at month 1, to 0.728 (0.667–0.788) at month 3 (*p* = 0.027, Fig. 5A). However, the mixed-model intercept 0.729 (0.623–0.836) overlapped the confidence interval of the pooled pre-DAT baseline mean 0.580 (0.519–0.636) for the randomization groups. For the more subjective EQ-VAS the mixed model indicated an effect of randomization (*F*[1, 36.4] = 4.52, *p* < 0.040) as well as of time post DAT (*F*[3, 18.8] = 6.45, *p* = 0.003). The *post-hoc* test showed a significant difference with higher EQ-VAS in the randomization group at 3 and 6 months (estimated mean difference +13.3 and +18.4, respectively), but not after 12 months (Fig 5B).

**Fig. 6:**
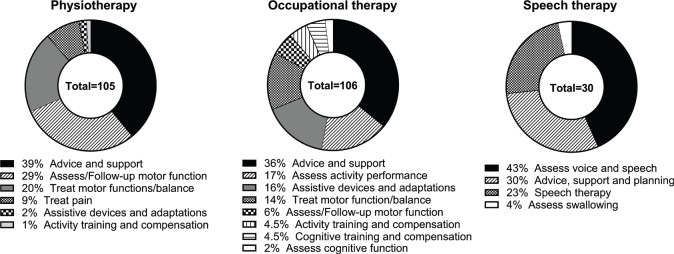
Distribution and volume of rehabilitation activities**.** Main reported activities in the standard care plus rehabilitation during the 3-month intervention.

Objective motor monitoring (PKG) showed no significant effects in linear mixed models, except a significant time effect for dyskinesia score and percentage time with immobility (see Table SI).

### Exploratory outcomes

The proportion of patients scoring below the AMPS motor threshold (< 1.5 logits) at baseline, 6, and 12 months was 14%, 14%, and 19% in SC+R vs 30%, 48%, and 54% in SC. For the process skills threshold (< 1.0 logits), proportions were 14%, 18%, and 10% in SC+R vs 9%, 13%, and 32% in SC. Exploratory longitudinal analysis of the proportion of patients scoring worse than threshold did not indicate a significant effect of randomization or interaction between time and randomization (data not shown), but there was a significant increase in the proportion of patients overall scoring worse than the ADL motor skill threshold over time (χ^2^ [2] = 7.953, *p* = 0.019 for the time factor).

## DISCUSSION

This is the first randomized controlled study of the effect of coordinated rehabilitation efforts following the initiation of DAT in PD on skills in activities of daily life, levels of participation, and HRQoL. There were no safety concerns, and the main finding was a difference between the standard care and rehabilitation groups regarding AMPS motor skill in favour of the rehabilitation intervention. The effect size (Cohen’s *d*) exceeded the medium size cut-off at the 6 months’ assessment that took place approximately 2 months after the rehabilitation period as well as 6 months later. The study was terminated early due to slow recruitment, partly due to the COVID-19 pandemic, so the intended sample size was not reached. Despite significant and clinically important differences in AMPS motor skill (16) at 6 and 12 months the study may have been underpowered with regard to showing effects on other outcome variables. The outcome is mainly consistent with a possible conserving effect of rehabilitation on abilities despite the progressive nature of Parkinson’s disease. Secondary findings include significantly better perceived health (EQ-VAS) in the intervention group at 3 and 6 months and confirmation of improved motor fluctuation as measured by MDS-UPDRS after the introduction of DAT (3, 4) as well as improved depression scores (MADRS-S) over time post DAT, but without indication of an effect of the rehabilitation intervention. Furthermore there was a positive development of cognition after rehabilitation. The effect size regarding cognition was, however, not clearly above a clinically important change (29).

There are few previous studies of rehabilitation after the initiation of DAT, and none of them is randomized. A retrospective observational study of postsurgical rehabilitation after STN-DBS indicated improvement of balance and gait performance in PD patients (30), but the same study could not show improvements in ADL ability as measured by the Barthel Index. This was in contrast to another open study which reported that rehabilitation after the STN-DBS operation improved both the UPDRS III motor score and the ADL scores Functional Independence Measure and Barthel Index (31). A recent Delphi study summarized the available evidence for rehabilitation after STN-DBS and cited only 12 observational studies, concluding that evidence was of low to moderate quality (12). The current randomized study is therefore an important contribution to the body of evidence regarding rehabilitation during a potential window for improvement.

Emerging therapy strategies for PD aim to improve neuroadaptation pharmacologically but there is also evidence for non-pharmacological therapies that support neuroplasticity such as cognitive training, and physical rehabilitation (32). A possible neuroprotective effect has also been attributed to rehabilitative activities (32, 33). Twelve months after DAT initiation, 19% of patients who underwent rehabilitation scored worse than the AMPS threshold of independence, compared with 54% of standard care patients. This represents a clinically meaningful pattern that supports further investigation. The observation suggests that a function-preserving effect of rehabilitation in this patient group could have practical importance for their autonomy and one possibility is that these effects reflect neuroplasticity.

A challenge in all rehabilitation studies is to ensure a sufficient dose of rehabilitation and to describe the content of rehabilitation, in particular when it is individualized. A network meta-analysis showed that 170 minutes of moderate-intensity physical activity per week corresponding to 40–45 min sessions, 4 times/week was needed for clinically meaningful improvement in people with PD and an optimal effect of dance was obtained with 60 min of dance 3–4 times/week (34). We used the billing codes for primary care rehabilitation in the region to assess dose. The advantage is consistent use of codes, but unfortunately the granularity of the codes is low. We can therefore only to some extent describe the interventions undertaken in rehabilitation sessions. The documented dose of rehabilitation in this study was low in relation to suggested dosing (34). Patients in the rehabilitation group had approximately 1h per week of supervised rehabilitation and the diary-reported activities indicated large variability between individuals regarding unsupervised activities. It is therefore probable that the rehabilitation was underdosed. Indeed, although outpatient management is generally preferred in PD, multidisciplinary rehabilitation may be better delivered as high intensity in-patient rehabilitation (35). Although this reflects the challenges of outpatient rehabilitation in this patient group, it is an important limitation regarding the effects on both primary and secondary outcomes in this study.

The rehabilitation interventions were coded as motor evaluation and training in 49% of sessions with physiotherapists and 20% with occupational therapists. Motor fluctuations are the core indication for DAT in PD patients, but non-motor symptoms become increasingly important as disease progresses and are not always improved with dopamine replacement therapy alone (36). The outcome measures of the present study consisted of a wide range of outcomes where motor skill and cognition were the only ones that differed between the groups. Fatigue, ADL, health-related quality of life, satisfaction, and experience of participation may all be influenced by both motor and non-motor symptoms and a focus mainly on motor aspects could have reduced the impact of rehabilitation. Assessment and cognitive training were only included by the OT and only coded in 7% of the total interventions, but there was nevertheless a significant difference in MoCA scores at 12 months. This could indicate some carry-over effects from other rehabilitation activities or activity in general, but an effect in the range 0.7 to 3.45 is not necessarily of clinical importance (29).

Strengths of this study include a very balanced randomization and that the intervention was made feasible within available healthcare services. It is also a strength that all DAT modalities available at the time were included and that the study was large enough to demonstrate that motor fluctuations improved after DAT initiation, although the lack of a control group without DAT formally precludes a firm conclusion regarding causality in that aspect. The original ambition was to include around 90 patients. For reasons including the intention to implement the study within the limitations of limited regular healthcare structures and the outbreak of the COVID-19 pandemic, the recruitment rate was too slow to achieve that within a reasonable time frame. The open design of the study inherently carries a risk of placebo effects that may exaggerate the intervention effect. Some indications of this are non-significantly better AMPS scores in the active intervention group already at baseline when they were aware of randomization outcome, as well as the intervention effect on the subjective EQ-VAS, but not on EQ5D5L-SI. It is also acknowledged that, due to the small numbers, several analyses are exploratory, and secondary outcomes were not corrected for multiplicity. This is a major limitation and together with some non-significant but clinically important differences this calls for further studies of rehabilitation intervention in association with DAT initiation.

In conclusion this randomized controlled trial provides preliminary evidence that coordinated rehabilitation following DAT initiation may help maintain ADL motor skills and may affect cognition positively. The findings support further investigation with a larger sample and can encourage clinicians to consider rehabilitation referral as part of standard post-DAT care.

## Supplementary Material



## References

[1] Norlin JM, Willis M, Persson U, Andersson E, S EP, Odin P. Swedish guidelines for device-aided therapies in Parkinson’s disease: economic evaluation and implementation. Acta Neurol Scand 2021; 144: 170–178. 10.1111/ane.13434 33899213

[2] Fasano A, Fung VSC, Lopiano L, Elibol B, Smolentseva IG, Seppi K, et al. Characterizing advanced Parkinson’s disease: OBSERVE-PD observational study results of 2615 patients. BMC Neurol 2019; 19: 50. 10.1186/s12883-019-1276-8 30940119 PMC6444751

[3] Deuschl G, Antonini A, Costa J, Smilowska K, Berg D, Corvol JC, et al. European Academy of Neurology/Movement Disorder Society-European Section Guideline on the Treatment of Parkinson’s Disease: I. Invasive Therapies. Mov Disord 2022; 37: 1360–1374. 10.1002/mds.29066 35791767

[4] de Bie RMA, Katzenschlager R, Swinnen B, Peball M, Lim SY, Mestre TA, et al. Update on treatments for Parkinson’s disease motor fluctuations: an international Parkinson and Movement Disorder Society evidence-based medicine review. Mov Disord 2025; 40: 776–794. 10.1002/mds.30162 40055961 PMC12089898

[5] Schupbach WM, Maltete D, Houeto JL, du Montcel ST, Mallet L, Welter ML, et al. Neurosurgery at an earlier stage of Parkinson disease: a randomized, controlled trial. Neurology 2007; 68: 267–271. 10.1212/01.wnl.0000250253.03919.fb 17151341

[6] Deuschl G, Agid Y. Subthalamic neurostimulation for Parkinson’s disease with early fluctuations: balancing the risks and benefits. Lancet Neurol 2013; 12: 1025–1034. 10.1016/S1474-4422(13)70151-0 24050735

[7] Schuepbach WM, Rau J, Knudsen K, Volkmann J, Krack P, Timmermann L, et al. Neurostimulation for Parkinson’s disease with early motor complications. N Engl J Med 2013; 368: 610–622. 10.1056/NEJMoa1205158 23406026

[8] Stoker V, Krack P, Tonder L, Barnett G, Durand-Zaleski I, Schnitzler A, et al. Deep brain stimulation impact on social and occupational functioning in Parkinson’s disease with early motor complications. Mov Disord Clin Pract 2020; 7: 672–680. 10.1002/mdc3.13015 32775513 PMC7396868

[9] Sahlstrom T, Eklund M, Timpka J, Henriksen T, Nyholm D, Odin P. Workforce participation and activities in Parkinson’s disease patients receiving device-aided therapy. Acta Neurol Scand 2018; 138: 78–84. 10.1111/ane.12929 29569237

[10] Hammell KW. Dimensions of meaning in the occupations of daily life. Can J Occup Ther 2004; 71: 296–305. 10.1177/000841740407100509 15633880

[11] Smilowska K, van Wamelen DJ, Pietrzykowski T, Calvano A, Rodriguez-Blazquez C, Martinez-Martin P, et al. Cost-effectiveness of device-aided therapies in Parkinson’s disease: a structured review. J Parkinsons Dis 2021; 11: 475–489. 10.3233/JPD-202348 33386813 PMC8150660

[12] Guidetti M, Marceglia S, Bocci T, Duncan R, Fasano A, Foote KD, et al. Is physical therapy recommended for people with Parkinson’s disease treated with subthalamic deep brain stimulation? A Delphi consensus study. J Neuroeng Rehabil 2025; 22: 80. 10.1186/s12984-025-01616-w 40211348 PMC11987424

[13] Hughes AJ, Daniel SE, Kilford L, Lees AJ. Accuracy of clinical diagnosis of idiopathic Parkinson’s disease: a clinico-pathological study of 100 cases. J Neurol Neurosurg Psychiatry 1992; 55: 181–184. 10.1136/jnnp.55.3.181 1564476 PMC1014720

[14] Sturkenboom IH, Graff MJ, Borm GF, Veenhuizen Y, Bloem BR, Munneke M, et al. The impact of occupational therapy in Parkinson’s disease: a randomized controlled feasibility study. Clin Rehabil 2013; 27: 99–112. 10.1177/0269215512448382 22811447

[15] Saghaei M. An overview of randomization and minimization programs for randomized clinical trials. J Med Signals Sens 2011; 1: 55–61. 22606659 PMC3317766

[16] Fisher A, Jones K. AMPS – Assessment of Motor and Process Skills. Volume 1: Development, standardization, and administration manual. Seventh, revised ed. Fort Collins, CO: Three Star Press; 2010.

[17] Fisher AG. The assessment of IADL motor skills: an application of many-faceted Rasch analysis. Am J Occup Ther 1993; 47: 319–329. 8322873 10.5014/ajot.47.4.319

[18] Hellström K, Lindmark B, Fugl-Meyer A. The Falls-Efficacy Scale, Swedish version: does it reflect clinically meaningful changes after stroke? Disabil Rehabil 2002; 24: 471–481. 10.1080/09638280110105259 12097216

[19] Johansson B, Starmark A, Berglund P, Rödholm M, Rönnbäck L. A self-assessment questionnaire for mental fatigue and related symptoms after neurological disorders and injuries. Brain Inj 2010; 24: 2–12. 10.3109/02699050903452961 20001478

[20] Cardol M, de Haan RJ, van den Bos GA, de Jong BA, de Groot IJ. The development of a handicap assessment questionnaire: the Impact on Participation and Autonomy (IPA). Clin Rehabil 1999; 13: 411–419. 10.1191/026921599668601325 10498348

[21] Cardol M, de Jong BA, van den Bos GA, Beelem A, de Groot IJ, de Haan RJ. Beyond disability: perceived participation in people with a chronic disabling condition. Clin Rehabil 2002; 16: 27–35. 10.1191/0269215502cr464oa 11841066

[22] Peto V, Jenkinson C, Fitzpatrick R. PDQ-39: a review of the development, validation and application of a Parkinson’s disease quality of life questionnaire and its associated measures. J Neurol 1998; 245: S10–S14. 10.1007/PL00007730 9617716

[23] Herdman M, Gudex C, Lloyd A, Janssen M, Kind P, Parkin D, et al. Development and preliminary testing of the new five-level version of EQ-5D (EQ-5D-5L). Qual Life Res 2011; 20: 1727–1736. 10.1007/s11136-011-9903-x 21479777 PMC3220807

[24] Goetz CG, Tilley BC, Shaftman SR, Stebbins GT, Fahn S, Martinez-Martin P, et al. Movement Disorder Society-sponsored revision of the Unified Parkinson’s Disease Rating Scale (MDS-UPDRS): scale presentation and clinimetric testing results. Mov Disord 2008; 23: 2129–2170. 10.1002/mds.22340 19025984

[25] Montgomery SA, Asberg M. A new depression scale designed to be sensitive to change. Br J Psychiatry 1979; 134: 382–389. 10.1192/bjp.134.4.382 444788

[26] Griffiths RI, Kotschet K, Arfon S, Xu ZM, Johnson W, Drago J, et al. Automated assessment of bradykinesia and dyskinesia in Parkinson’s disease. J Parkinsons Dis 2012; 2: 47–55. 10.3233/JPD-2012-11071 23939408

[27] Chaudhuri KR, Yates L, Martinez-Martin P. The non-motor symptom complex of Parkinson’s disease: a comprehensive assessment is essential. Curr Neurol Neurosci Rep 2005; 5: 275–283. 10.1007/s11910-005-0072-6 15987611

[28] Nasreddine ZS, Phillips NA, Bedirian V, Charbonneau S, Whitehead V, Collin I, et al. The Montreal Cognitive Assessment, MoCA: a brief screening tool for mild cognitive impairment. J Am Geriatr Soc 2005; 53: 695–699. 10.1111/j.1532-5415.2005.53221.x 15817019

[29] Lindvall E, Abzhandadze T, Quinn TJ, Sunnerhagen KS, Lundstrom E. Is the difference real, is the difference relevant: the minimal detectable and clinically important changes in the Montreal Cognitive Assessment. Cereb Circ Cogn Behav 2024; 6: 100222. 10.1016/j.cccb.2024.100222 38745691 PMC11090903

[30] Sasaki F, Sekimoto S, Jo T, Hatano T, Shimo Y, Iwamuro H, et al. Balance and gait improvements of postoperative rehabilitation in patients with Parkinson’s disease treated with subthalamic nucleus deep brain stimulation (STN-DBS). Parkinson’s Dis 2019; 2019: 1–5. 10.1155/2019/7104071 PMC6701295 31467660

[31] Tassorelli C, Buscone S, Sandrini G, Pacchetti C, Furnari A, Zangaglia R, et al. The role of rehabilitation in deep brain stimulation of the subthalamic nucleus for Parkinson’s disease: a pilot study. Park Relat Disord 2009; 15: 675–681. 10.1016/j.parkreldis.2009.03.006 19398215

[32] Kopalli SR, Behl T, Baldaniya L, Ballal S, Joshi KK, Arya R, et al. Neuroadaptation in neurodegenerative diseases: compensatory mechanisms and therapeutic approaches. Prog Neuro-psychopharmacol Biol Psychiatry 2025; 139: 111375. 10.1016/j.pnpbp.2025.111375 40280271

[33] Chromiec PA, Urbaś ZK, Jacko M, Kaczor JJ. The proper diet and regular physical activity slow down the development of Parkinson disease. Aging Dis 2021; 12: 1605–1623. 10.14336/AD.2021.0123 34631210 PMC8460298

[34] Zheng S, Yu Y, Liang S, Hou W, Hu X, Wu F, et al. Effects of tele-rehabilitation after deep brain stimulation on cardiopulmonary fitness and gait in patients with Parkinson’s disease. Neurol Sci 2025; 46: 5073–5080. 10.1007/s10072-025-08222-7 40407994

[35] Radder DLM, Ligia Silva de Lima A, Domingos J, Keus SHJ, van Nimwegen M, Bloem BR, et al. Physiotherapy in Parkinson’s disease: a meta-analysis of present treatment modalities. Neurorehabil Neural Repair 2020; 34: 871–880. 10.1177/1545968320952799 32917125 PMC7564288

[36] Kamel WA, Al-Hashel JY. LCIG in treatment of non-motor symptoms in advanced Parkinson’s disease: review of literature. Brain Behav 2020; 10: e01757–n/a. 10.1002/brb3.1757 32677345 PMC7507541

