# Supplementary material for: OUTCOMES OF REHABILITATION FOLLOWING DEVICE-AIDED THERAPY START IN PARKINSON’S DISEASE: A RANDOMIZED CONTROLLED TRIAL
Source: J Rehabil Med. 2026 Aug 3;58:46068. doi: 10.2340/jrm.v58.46068 (PMC13449224; doi:10.2340/jrm.v58.46068)
Supplement: Supplementary file 1 [file JRM-58-46068-s1.pdf]

Table SI. Summary of main outcomes of linear mixed models for other outcome measures

**Outcome measure**	**Effect**	**Num df**	**Denom df**	***F***	***p-value***
AMPS Process Skills	Rehabilitation	1	43.1	0.02	0.90
	Time	1	42.5	1.22	0.28
	Interaction	1	42.5	3.97	0.05
IPA Autonomy Indoors	Rehabilitation	1	43.3	0.16	0.90
	Time	1	42.8	1.08	0.31
	Interaction	1	42.8	1.16	0.29
IPA Autonomy Outdoors	Rehabilitation	1	43.2	2.61	0.11
	Time	1	42.7	0.23	0.64
	Interaction	1	42.7	0.23	0.64
IPA Family Role	Rehabilitation	1	43.2	0.06	0.37
	Time	1	42.9	3.89	0.06
	Interaction	1	42.9	2.37	0.131
IPA Social Life	Rehabilitation	1	43.1	0.06	0.82
	Time	1	42.4	0.27	0.61
	Interaction	1	42.4	1.69	0.20
IPA Work	Rehabilitation	1	8.0	0.17	0.69
	Time	1	8.0	0.17	0.69
	Interaction	1	8.0	0.08	0.79
Falls Efficacy Scale, personal ADL (Item 1-6)	Rehabilitation	1	43.2	0.00	0.96
	Time	1	42.3	2.40	0.13
	Interaction	1	42.3	1.70	0.20
Falls Efficacy Scale, instrumental ADL (Item 8-13)	Rehabilitation	1	43.2	0.05	0.82
	Time	1	42.7	1.55	0.22
	Interaction	1	42.7	3.07	0.06
Falls Efficacy Scale, total score (Item 1-13)	Rehabilitation	1	43.1	0.02	0.90
	Time	1	42.5	1.44	0.24
	Interaction	1	42.5	3.60	0.07
Mental Fatigue Scale total score (item 1-14)	Rehabilitation	1	43.1	0.34	0.56
	Time	1	42.7	0.02	0.90
	Interaction	1	42.7	1.46	0.23
PKG BK-median score (BKS)	Rehabilitation	1	27.0	0.58	0.45
	Time	2	11.6	0.20	0.82
	Interaction	2	11.6	1.11	0.36
PKG DK-median score (DKS)	Rehabilitation	1	28.1	0.01	0.92
	Time	2	10.8	6.66	0.01
	Interaction	2	10.8	1.28	0.32

Outcome measure	Effect	Num df	Denom df	*F*	*p-value*
PKG Fluctuation Dyskinesia Score (FDS)	Rehabilitation	1	28.4	0.05	0.82
	Time	2	15.9	2.04	0.16
	Interaction	2	15.9	0.36	0.70
PKG Percent time with tremor (PTT)	Rehabilitation	1	18.2	0.31	0.58
	Time	2	7.4	1.22	0.35
	Interaction	2	7.4	1.85	0.22
PKG Percent time in immobility (PTI)	Rehabilitation	1	8.7	2.29	0.17
	Time	2	6.9	5.80	0.03
	Interaction	2	6.9	0.47	0.64

AMPS: Assessment of Motor and Process Skills; IPA: Impact of Participation and Autonomy; PKG: Parkinson Kinetigraph; BK: Bradykinesia Score; DK: Dyskinesia Score; FDS: Fluctuation Dyskinesia Score; PTT: Percent Time with Tremor; PTI: Percent Time with Immobility
